# The genome sequence of the flutter-wing fly, *Palloptera scutellata *(Macquart, 1835)

**DOI:** 10.12688/wellcomeopenres.20381.1

**Published:** 2023-11-20

**Authors:** Michael Ashworth, Duncan Sivell, Natural History Museum Genome Acquisition Lab, Darwin Tree of Life Barcoding collective, Wellcome Sanger Institute Tree of Life programme, Wellcome Sanger Institute Scientific Operations: DNA Pipelines collective, Tree of Life Core Informatics collective, Darwin Tree of Life Consortium

**Affiliations:** 1 Independent researcher, Yeovil, England, UK; 2 Natural History Museum, London, England, UK

**Keywords:** Palloptera scutellata, flutter-wing fly, genome sequence, chromosomal, Diptera

## Abstract

We present a genome assembly from an individual female *Palloptera scutellata* (the flutter-wing fly; Arthropoda; Insecta; Diptera; Pallopteridae). The genome sequence is 415.6 megabases in span. Most of the assembly is scaffolded into 5 chromosomal pseudomolecules. The mitochondrial genome has also been assembled and is 15.93 kilobases in length.

## Species taxonomy

Eukaryota; Metazoa; Eumetazoa; Bilateria; Protostomia; Ecdysozoa; Panarthropoda; Arthropoda; Mandibulata; Pancrustacea; Hexapoda; Insecta; Dicondylia; Pterygota; Neoptera; Endopterygota; Diptera; Brachycera; Muscomorpha; Eremoneura; Cyclorrhapha; Schizophora; Acalyptratae; Tephritoidea; Pallopteridae; *Palloptera*; *Palloptera scutellata* (Macquart, 1835) (NCBI:txid2849646).

## Background

The Pallopteridae is a family of small to medium sized (2.5–7 mm), acalypterate flies sometimes known as the flutter-wing or trembling-wing flies, because of the rapid wing movements made by many species. The family is identified by the following characters: subcosta complete and curving smoothly to end in the costa, a costal break at the junction of the subcosta, first radial vein (R _1_) bare on the dorsal surface, head with one pair of reclinate orbital setae, postvertical bristles parallel to divergent, vibrissae absent, and wings usually with markings consisting of clouded areas and spots. On account of their patterns of wing spots, in the British fauna, the Pallopteridae – along with the families Tephritidae, Opomyzidae, Platystomatidae and Ulidiidae – are often known collectively as the picture-winged flies. There are around 83 described species of the Pallopteridae in 15 genera which are found in the temperate regions of the globe ( GBIF Secretariat, 2023).

With 46 species, the genus *Palloptera* contains the majority of the Pallopteridae. *Palloptera scutellata* (Macquart, 1835) (Diptera: Pallopteridae) has orange-brown abdomen and legs, a blue-grey thorax and an orange frons. It is readily identified by the wing pattern with four spots, comprising the wing stigma in the subcostal cell, two small patches covering the anterior and posterior crossveins, and a shaded area towards the wing tip but not including the wing apex, which is clear.

*P. scutellata* is found in northern Europe with most records in Great Britain and Northern Ireland, Germany, the Netherlands and Belgium. Females have a long piercing ovipositor and lay eggs in the stems of the Soft Rush, *Juncus effusus*, where the larvae are phytophagous ( Rotheray & Hewitt, 2015). The life cycle is unusual within the genus with females emerging in the autumn and overwintering in the mated state ( Bland & Horsfield, 2016). This life cycle is distinct from that of other European species of *Palloptera*, in which larvae are the overwintering stage ( Rotheray, 2014).

In the United Kingdom, *Palloptera scutellata* was first recorded in 1950 at Common, Surrey ( Parmenter, 1950). Subsequent records show a widespread but scattered distribution across England and Wales. It was first recorded in Scotland as recently as 2015 but has been found to be widespread ( Bland & Horsfield, 2016). The presence of the species in Ireland was noted by Speight (1979), and Smit *et al.* (2009) reported the species as new to Belgium. This flurry of new announcements is indicative of a fly whose populations are thriving and increasing in range. Nevertheless, it is found much less frequently than its host plant implying that it may have special requirements, as yet unknown, but probably including warm, sheltered locations.

A female *Palloptera scutellata* was taken on 15 May 2021 at Stover Country Park, South Devon in the south-west of England during a meeting of the Devon Fly Group, a local group of the Dipterists Forum, the UK’s national society for the study of flies. The specimen was sent live to the Natural History Museum, London. Stover Country Park is a Local Nature Reserve and designated as a Site of Special Scientific Interest. The site comprises 46 hectares of mixed habitats including woodland, lake, marsh, heathland, and grassland.

The generation of a high-quality genome sequence for *Palloptera scutellata* is an important step in advancing the understanding of these fascinating flies and in determining relationships between species within the genus *Palloptera* and within the Pallopteridae.

## Genome sequence report

**Figure 1.**
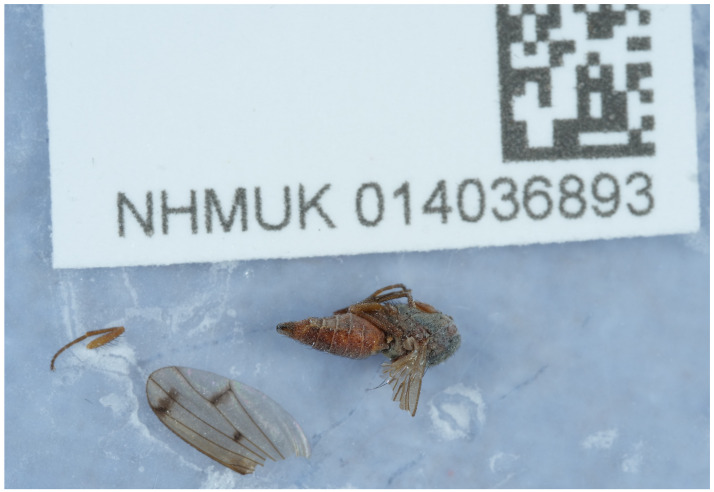
Photograph of the *Palloptera scutellata* (idPalScut3) specimen used for genome sequencing.

The genome was sequenced from one female *Palloptera scutellata* ( Figure 1) collected from Stover Country Park, England (50.57, –3.65). A total of 46-fold coverage in Pacific Biosciences single-molecule HiFi long reads was generated. Primary assembly contigs were scaffolded with chromosome conformation Hi-C data. Manual assembly curation corrected 50 missing joins or mis-joins, reducing the scaffold number by 24.14%, and increasing the scaffold N50 by 0.58%.

**Figure 2.**
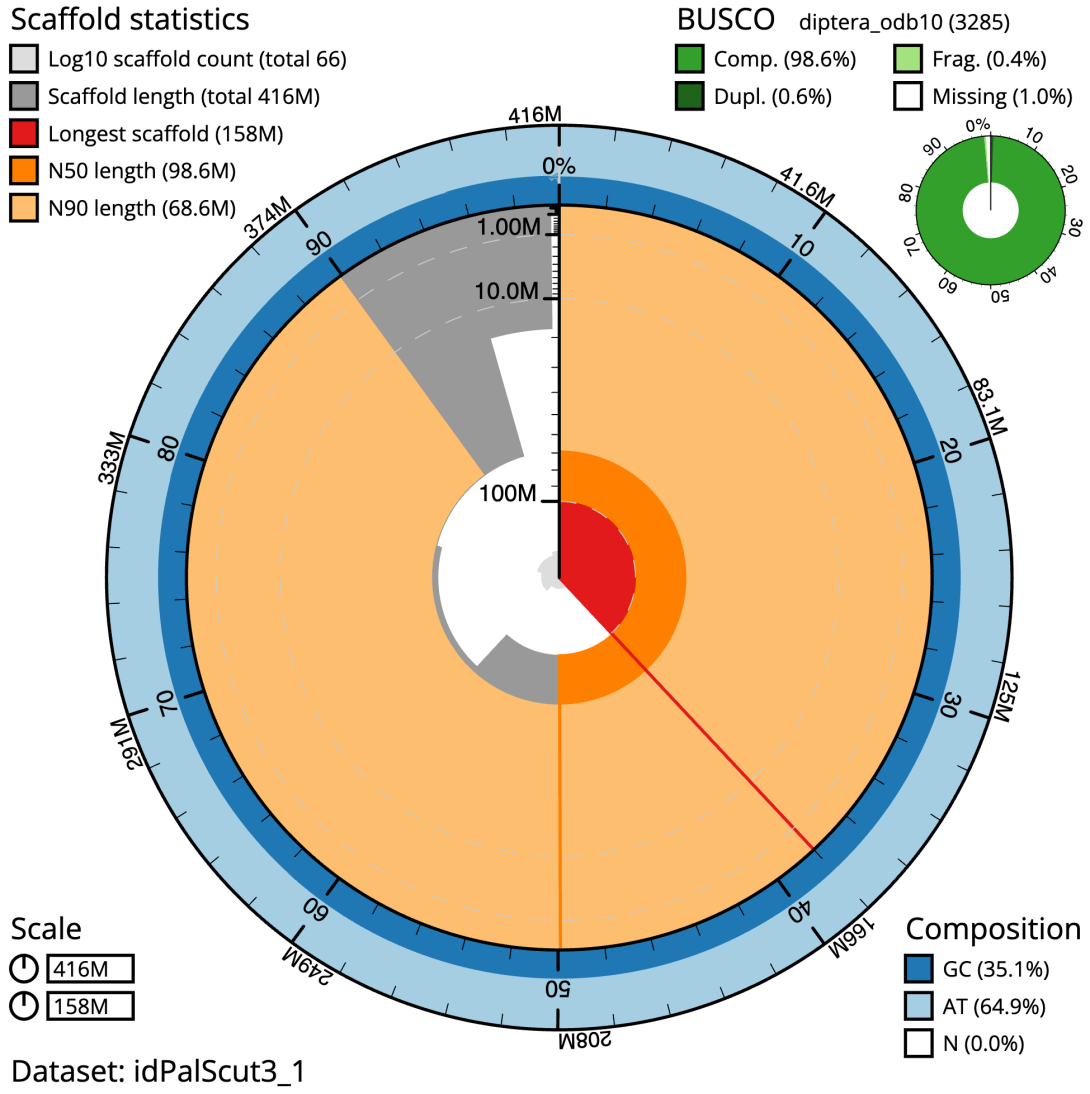
Genome assembly of *Palloptera scutellata*, idPalScut3.1: metrics. The BlobToolKit Snailplot shows N50 metrics and BUSCO gene completeness. The main plot is divided into 1,000 size-ordered bins around the circumference with each bin representing 0.1% of the 415,657,720 bp assembly. The distribution of scaffold lengths is shown in dark grey with the plot radius scaled to the longest scaffold present in the assembly (158,007,244 bp, shown in red). Orange and pale-orange arcs show the N50 and N90 scaffold lengths (98,550,696 and 68,594,659 bp), respectively. The pale grey spiral shows the cumulative scaffold count on a log scale with white scale lines showing successive orders of magnitude. The blue and pale-blue area around the outside of the plot shows the distribution of GC, AT and N percentages in the same bins as the inner plot. A summary of complete, fragmented, duplicated and missing BUSCO genes in the diptera_odb10 set is shown in the top right. An interactive version of this figure is available at https://blobtoolkit.genomehubs.org/view/Palloptera%20scutellata/dataset/idPalScut3_1/snail.

**Figure 3.**
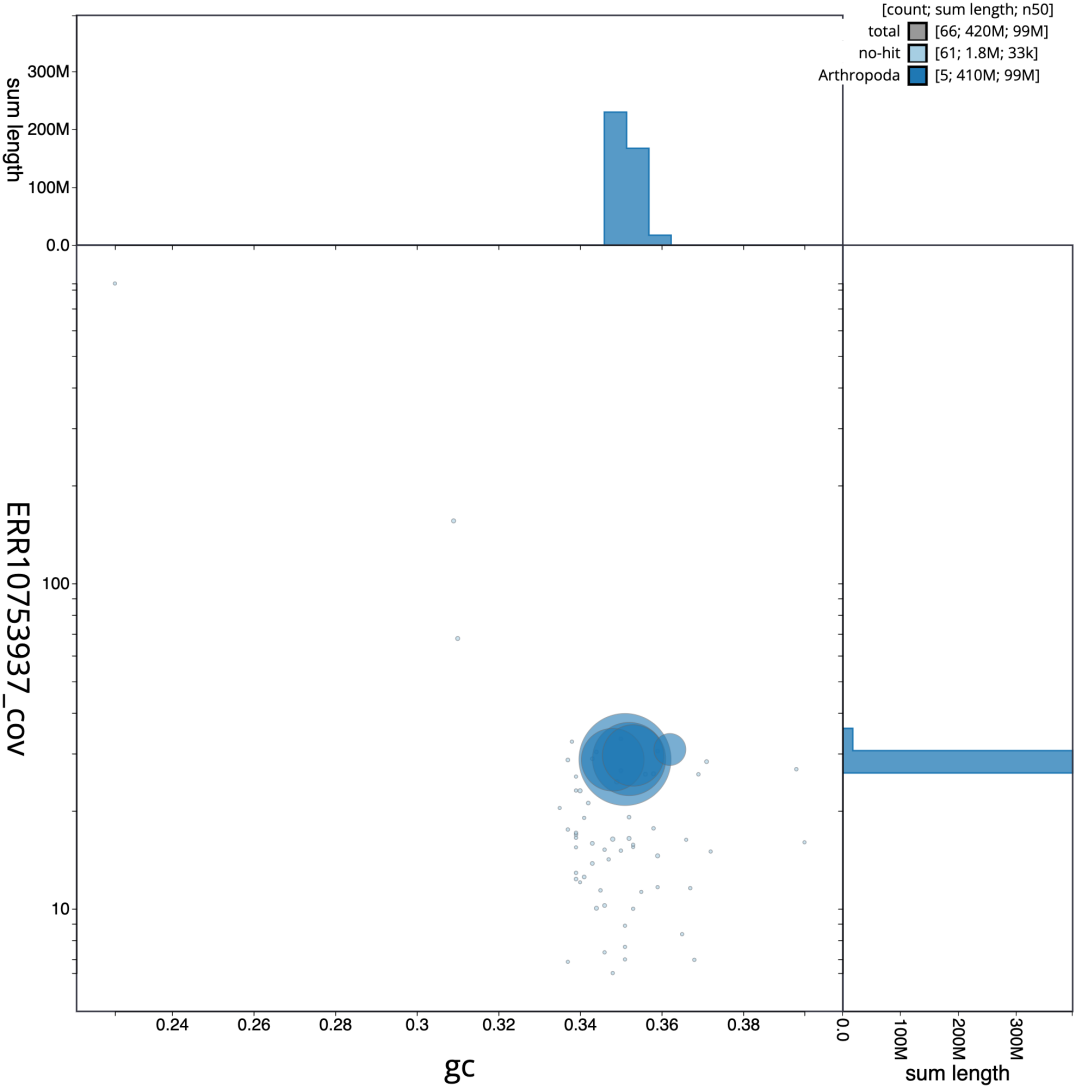
Genome assembly of *Palloptera scutellata*, idPalScut3.1: BlobToolKit GC-coverage plot. Scaffolds are coloured by phylum. Circles are sized in proportion to scaffold length. Histograms show the distribution of scaffold length sum along each axis. An interactive version of this figure is available at https://blobtoolkit.genomehubs.org/view/Palloptera%20scutellata/dataset/idPalScut3_1/blob.

**Figure 4.**
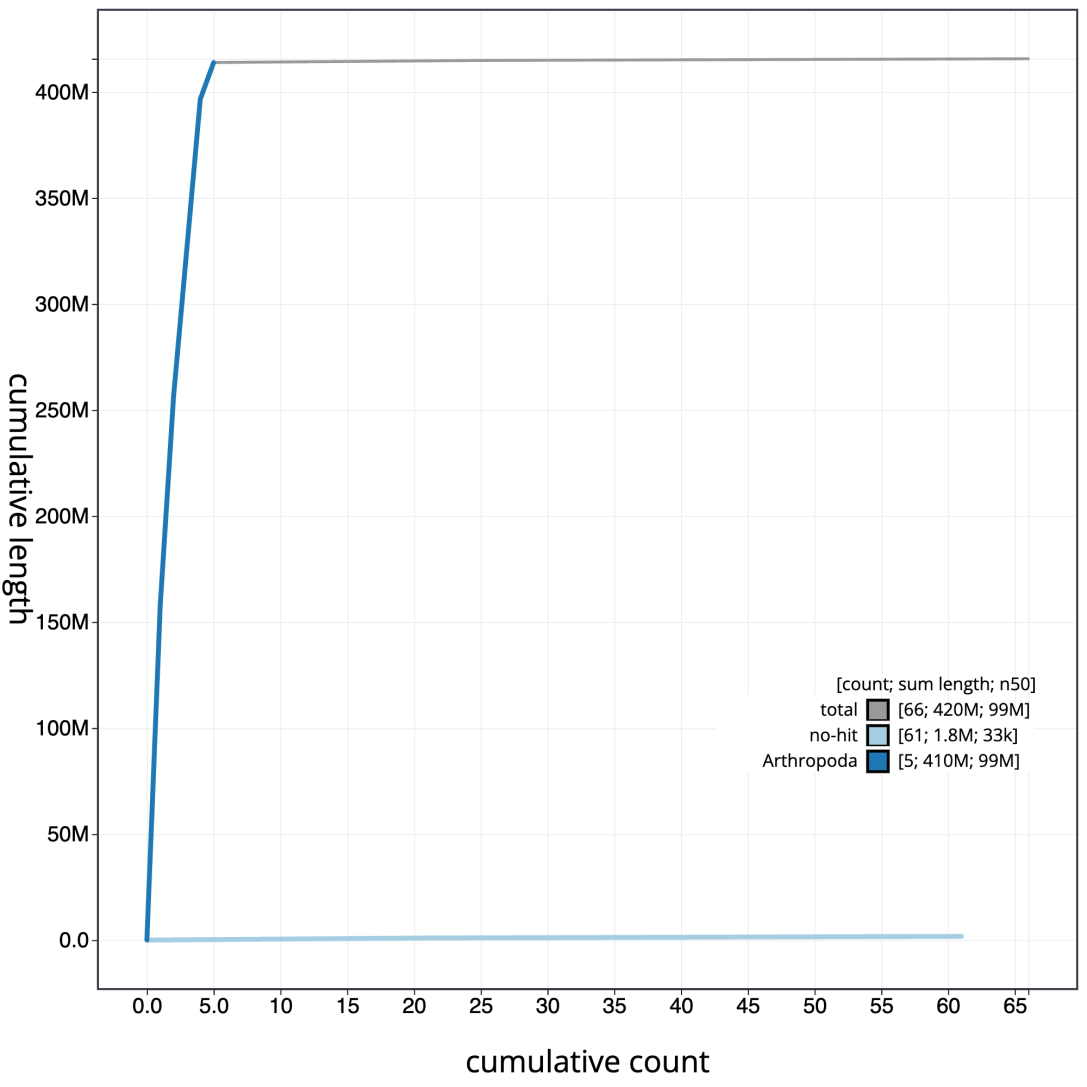
Genome assembly of *Palloptera scutellata*, idPalScut3.1: BlobToolKit cumulative sequence plot. The grey line shows cumulative length for all scaffolds. Coloured lines show cumulative lengths of scaffolds assigned to each phylum using the buscogenes taxrule. An interactive version of this figure is available at https://blobtoolkit.genomehubs.org/view/Palloptera%20scutellata/dataset/idPalScut3_1/cumulative.

**Figure 5.**
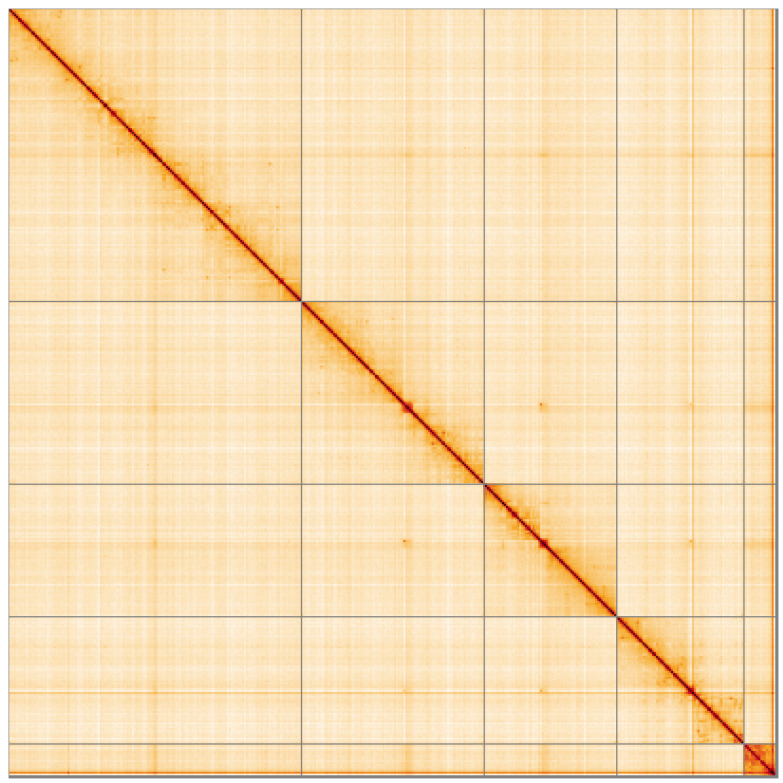
Genome assembly of *Palloptera scutellata*, idPalScut3.1: Hi-C contact map of the idPalScut3.1 assembly, visualised using HiGlass. Chromosomes are shown in order of size from left to right and top to bottom. An interactive version of this figure may be viewed at https://genome-note-higlass.tol.sanger.ac.uk/l/?d=LehCkdUsR3u6lpCbO5ESsA.

The final assembly has a total length of 415.6 Mb in 65 sequence scaffolds with a scaffold N50 of 98.6 Mb ( Table 1). The snailplot in Figure 2 provides a summary of the assembly statistics, while the distribution of assembly scaffolds on GC proportion and coverage is shown in Figure 3. The cumulative assembly plot in Figure 4 shows curves for subsets of scaffolds assigned to different phyla. Most (99.57%) of the assembly sequence was assigned to 5 chromosomal-level scaffolds. We did not identify the sex chromosome as sequence data from the heterogametic sex was not available, and homology is unreliable for sex chromosome identification in Diptera due to frequent sex chromosome turnover ( Vicoso & Bachtrog, 2015). Chromosome-scale scaffolds confirmed by the Hi-C data are named in order of size ( Figure 5; Table 2). While not fully phased, the assembly deposited is of one haplotype. Contigs corresponding to the second haplotype have also been deposited. The mitochondrial genome was also assembled and can be found as a contig within the multifasta file of the genome submission.

**Table 1.** Genome data for *Palloptera scutellata*, idPalScut3.1.

Project accession data		
Assembly identifier	idPalScut3.1	
Assembly release date	2023-07-06	
Species	*Palloptera scutellata*	
Specimen	idPalScut3	
NCBI taxonomy ID	2849646	
BioProject	PRJEB58669	
BioSample ID	SAMEA14448439	
Isolate information	idPalScut3, female: thorax (DNA sequencing) idPalScut1: head and thorax (Hi-C data)	
Assembly metrics *		Benchmark
Consensus quality (QV)	60.3	*≥ 50*
*k*-mer completeness	100%	*≥ 95%*
BUSCO **	C:98.6%[S:98.0%,D:0.6%],F:0.4%, M:1.0%,n:3,285	*C ≥ 95%*
Percentage of assembly mapped to chromosomes	99.57%	*≥ 95%*
Sex chromosomes	Not identified	*localised homologous pairs*
Organelles	Mitochondrial genome assembled	*complete single alleles*
Raw data accessions		
PacificBiosciences SEQUEL II	ERR10753937	
Hi-C Illumina	ERR10742415	
Genome assembly		
Assembly accession	GCA_958295655.1	
*Accession of alternate haplotype*	GCA_958295705.1	
Span (Mb)	415.6	
Number of contigs	389	
Contig N50 length (Mb)	2.5	
Number of scaffolds	65	
Scaffold N50 length (Mb)	98.6	
Longest scaffold (Mb)	158	

* Assembly metric benchmarks are adapted from column VGP-2020 of “Table 1: Proposed standards and metrics for defining genome assembly quality” from ( Rhie *et al.*, 2021). ** BUSCO scores based on the diptera_odb10 BUSCO set using v5.3.2. C = complete [S = single copy, D = duplicated], F = fragmented, M = missing, n = number of orthologues in comparison. A full set of BUSCO scores is available at https://blobtoolkit.genomehubs.org/view/Palloptera%20scutellata/dataset/idPalScut3_1/busco.

**Table 2.** Chromosomal pseudomolecules in the genome assembly of *Palloptera scutellata*, idPalScut3.

INSDC accession	Chromosome	Length (Mb)	GC%
OY282449.1	1	158.01	35.0
OY282450.1	2	98.55	35.0
OY282451.1	3	71.52	35.0
OY282452.1	4	68.59	35.5
OY282453.1	5	17.21	36.0
OY282454.1	MT	0.02	22.5

The estimated Quality Value (QV) of the final assembly is 60.3 with *k*-mer completeness of 100%, and the assembly has a BUSCO v5.3.2 completeness of 98.6% (single = 98.0%, duplicated = 0.6%), using the diptera_odb10 reference set ( *n* = 3,285).

Metadata for specimens, barcode results, spectra estimates, sequencing runs, contaminants and pre-curation assembly statistics are given at https://links.tol.sanger.ac.uk/species/2849646

## Methods

### Sample acquisition and nucleic acid extraction

The specimen used for genome sequencing was a female *Palloptera scutellata* (specimen ID NHMUK014036893, ToLID idPalScut3) collected using an aerial net from Stover Country Park, England (latitude 50.57, longitude –3.65) on 2021-05-15. The specimen was collected and identified by Michael Ashworth (independent researcher) and then dry frozen (–80°C). The specimen used for Hi-C sequencing (specimen ID NHMUK014449035, ToLID idPalScut1) was collected using an aerial net from Bookham Common on 2021-04-20. The specimen was collected and identified by Duncan Sivell (Natural History Museum) and preserved in ethanol.

The workflow for high molecular weight (HMW) DNA extraction at the Wellcome Sanger Institute (WSI) includes a sequence of core procedures: sample preparation; sample homogenisation; DNA extraction; HMW DNA fragmentation; and fragmented DNA clean-up. The sample was prepared for DNA extraction at the WSI Tree of Life laboratory: the idPalScut3 sample was weighed and dissected on dry ice with tissue set aside for Hi-C sequencing ( https://dx.doi.org/10.17504/protocols.io.x54v9prmqg3e/v1). Tissue from the thorax was disrupted using a Nippi Powermasher fitted with a BioMasher pestle ( https://dx.doi.org/10.17504/protocols.io.5qpvo3r19v4o/v1). DNA was extracted at the WSI Scientific Operations core using the Qiagen MagAttract HMW DNA kit, according to the manufacturer’s instructions.

All protocols used by the Tree of Life laboratory are publicly available on protocols.io ( https://dx.doi.org/10.17504/protocols.io.8epv5xxy6g1b/v1).

### Sequencing

Pacific Biosciences HiFi circular consensus DNA sequencing libraries were constructed according to the manufacturers’ instructions. DNA sequencing was performed by the Scientific Operations core at the WSI on a Pacific Biosciences SEQUEL II (HiFi) instrument. Hi-C data were also generated from head and thorax tissue of idPalScut1 using the Arima2 kit and sequenced on the Illumina NovaSeq 6000 instrument.

### Genome assembly, curation and evaluation

Assembly was carried out with Hifiasm ( Cheng *et al.*, 2021) and haplotypic duplication was identified and removed with purge_dups ( Guan *et al.*, 2020). The assembly was then scaffolded with Hi-C data ( Rao *et al.*, 2014) using YaHS ( Zhou *et al.*, 2023). The assembly was checked for contamination and corrected as described previously ( Howe *et al.*, 2021). Manual curation was performed using HiGlass ( Kerpedjiev *et al.*, 2018) and Pretext ( Harry, 2022). The mitochondrial genome was assembled using MitoHiFi ( Uliano-Silva *et al.*, 2023), which runs MitoFinder ( Allio *et al.*, 2020) or MITOS ( Bernt *et al.*, 2013) and uses these annotations to select the final mitochondrial contig and to ensure the general quality of the sequence.

A Hi-C map for the final assembly was produced using bwa-mem2 ( Vasimuddin *et al.*, 2019) in the Cooler file format ( Abdennur & Mirny, 2020). To assess the assembly metrics, the *k*-mer completeness and QV consensus quality values were calculated in Merqury ( Rhie *et al.*, 2020). This work was done using Nextflow ( Di Tommaso *et al.*, 2017) DSL2 pipelines “sanger-tol/readmapping” ( Surana *et al.*, 2023a) and “sanger-tol/genomenote” ( Surana *et al.*, 2023b). The genome was analysed within the BlobToolKit environment ( Challis *et al.*, 2020) and BUSCO scores ( Manni *et al.*, 2021; Simão *et al.*, 2015) were calculated.

Table 3 contains a list of relevant software tool versions and sources.

**Table 3.** Software tools: versions and sources.

Software tool	Version	Source
BlobToolKit	4.2.1	https://github.com/blobtoolkit/blobtoolkit
BUSCO	5.3.2	https://gitlab.com/ezlab/busco
Hifiasm	0.16.1-r375	https://github.com/chhylp123/hifiasm
HiGlass	1.11.6	https://github.com/higlass/higlass
Merqury	MerquryFK	https://github.com/thegenemyers/MERQURY.FK
MitoHiFi	2	https://github.com/marcelauliano/MitoHiFi
PretextView	0.2	https://github.com/wtsi-hpag/PretextView
purge_dups	1.2.3	https://github.com/dfguan/purge_dups
sanger-tol/genomenote	v1.0	https://github.com/sanger-tol/genomenote
sanger-tol/readmapping	1.1.0	https://github.com/sanger-tol/readmapping/tree/1.1.0
YaHS	1.2a	https://github.com/c-zhou/yahs

### Wellcome Sanger Institute – Legal and Governance

The materials that have contributed to this genome note have been supplied by a Darwin Tree of Life Partner. The submission of materials by a Darwin Tree of Life Partner is subject to the **‘Darwin Tree of Life Project Sampling Code of Practice’**, which can be found in full on the Darwin Tree of Life website here. By agreeing with and signing up to the Sampling Code of Practice, the Darwin Tree of Life Partner agrees they will meet the legal and ethical requirements and standards set out within this document in respect of all samples acquired for, and supplied to, the Darwin Tree of Life Project.

Further, the Wellcome Sanger Institute employs a process whereby due diligence is carried out proportionate to the nature of the materials themselves, and the circumstances under which they have been/are to be collected and provided for use. The purpose of this is to address and mitigate any potential legal and/or ethical implications of receipt and use of the materials as part of the research project, and to ensure that in doing so we align with best practice wherever possible. The overarching areas of consideration are:

•   Ethical review of provenance and sourcing of the material

•   Legality of collection, transfer and use (national and international)

Each transfer of samples is further undertaken according to a Research Collaboration Agreement or Material Transfer Agreement entered into by the Darwin Tree of Life Partner, Genome Research Limited (operating as the Wellcome Sanger Institute), and in some circumstances other Darwin Tree of Life collaborators.

## Data Availability

European Nucleotide Archive: *Palloptera scutellata* Accession number PRJEB58669; https://identifiers.org/ena.embl/PRJEB58669 ( Wellcome Sanger Institute, 2023). The genome sequence is released openly for reuse. The *Palloptera scutellata* genome sequencing initiative is part of the Darwin Tree of Life (DToL) project. All raw sequence data and the assembly have been deposited in INSDC databases. The genome will be annotated using available RNA-Seq data and presented through the Ensembl pipeline at the European Bioinformatics Institute. Raw data and assembly accession identifiers are reported in Table 1.

## Author information

Members of the Natural History Museum Genome Acquisition Lab are listed here: https://doi.org/10.5281/zenodo.7139035.

Members of the Darwin Tree of Life Barcoding collective are listed here: https://doi.org/10.5281/zenodo.4893703.

Members of the Wellcome Sanger Institute Tree of Life programme are listed here: https://doi.org/10.5281/zenodo.4783585.

Wellcome Sanger Institute Scientific Operations: Sequencing Operations author list are listed here: https://doi.org/10.5281/zenodo.10043364.

Members of the Tree of Life Core Informatics collective are listed here: https://doi.org/10.5281/zenodo.5013541.

Members of the Darwin Tree of Life Consortium are listed here: https://doi.org/10.5281/zenodo.4783558.
