# Supplementary material for: The genome sequence of the flutter-wing fly, Palloptera scutellata (Macquart, 1835)
Source: Wellcome Open Res. 2023 Nov 20;8:534. [Version 1] doi: 10.12688/wellcomeopenres.20381.1 (PMC11126900; doi:10.12688/wellcomeopenres.20381.1)
Supplement: Peer review history

# Reviewer report: Reviewer response for version 1

**DOI:** 10.21956/wellcomeopenres.22568.r71314

**Published:** 2024-05-25

**Authors:** Jerome H.L Hui

**Affiliations:** 1 Chinese University of Hong Kong, Hong Kong, China

Ashworth and colleagues report the genome sequence of fly *Palloptera scutellata *(Macquart 1835). According to my own search, there is currently no molecular data of this species being available in the public domain such as the NCBI database (before this report). This newly generated genomic resource serves as an important foundation to further studies, such as revealing if there are any cryptic species, understanding their population genetic/genomic structure, asking evolutionary and genomics questions related to dipterans and insects more widely, as well as aid in species identification (as citizen science project for example).

 This genome resource is excellent from the summary statistics, with high BUSCO numbers, high sequence continuity (scaffold N50), and majority of sequences contained on the 5 pseudochromosomes (plus mitochondrion). To sum up, this is a valuable contribution setting up a nice foundation for further studies.

Are sufficient details of methods and materials provided to allow replication by others?

Yes

Is the rationale for creating the dataset(s) clearly described?

Yes

Are the datasets clearly presented in a useable and accessible format?

Yes

Are the protocols appropriate and is the work technically sound?

Yes

Reviewer Expertise:

Genomics, evolution, invertebrates

I confirm that I have read this submission and believe that I have an appropriate level of expertise to confirm that it is of an acceptable scientific standard.

# Reviewer report: Reviewer response for version 1

**DOI:** 10.21956/wellcomeopenres.22568.r71309

**Published:** 2024-02-02

**Authors:** Shu-Jun Wei

**Affiliations:** 1 Beijing Academy of Agriculture and Forestry Sciences, Beijing, China

Michael Ashworth and Duncan Sivell have assembled the nuclear and mitochondrial genomes of the flutter-wing fly, *Palloptera scutellata* (Diptera; Pallopteridae) using Pacific Biosciences single-molecule HiFi long reads and chromosome conformation Hi-C data. The final assembly has a total length of 415.6 Mb and a BUSCO completeness of 98.6%. This high-quality genome is a valuable resource for understanding the genetics and evolution of this fly and other dipterans. However, it should be noted that this genome assembly has not yet been annotated.

Are sufficient details of methods and materials provided to allow replication by others?

Yes

Is the rationale for creating the dataset(s) clearly described?

Yes

Are the datasets clearly presented in a useable and accessible format?

Yes

Are the protocols appropriate and is the work technically sound?

Yes

Reviewer Expertise:

Population genetics, genomics, and pest control.

I confirm that I have read this submission and believe that I have an appropriate level of expertise to confirm that it is of an acceptable scientific standard.
